# Protein Intakes during Weaning from Parenteral Nutrition Drive Growth Gain and Body Composition in Very Low Birth Weight Preterm Infants

**DOI:** 10.3390/nu12051298

**Published:** 2020-05-02

**Authors:** Nadia Liotto, Orsola Amato, Pasqua Piemontese, Camilla Menis, Anna Orsi, Maria Grazia Corti, Mariarosa Colnaghi, Valeria Cecchetti, Lorenza Pugni, Fabio Mosca, Paola Roggero

**Affiliations:** 1 Neonatal Intensive Care Unit, Fondazione IRCCS Ca’ Granda Ospedale Maggiore Policlinico, 20122 Milan, Italy; nadia.liotto@policlinico.mi.it (N.L.); orsola.amato@policlinico.mi.it (O.A.); pasqua.piemontese@policlinico.mi.it (P.P.); camilla.menis@gmail.com (C.M.); anna.orsi@policlinico.mi.it (A.O.); mariarosa.colnaghi@policlinico.mi.it (M.C.); valeria.cecchetti@mangiagalli.it (V.C.); lorenza.pugni@mangiagalli.it (L.P.); fabio.mosca@unimi.it (F.M.); 2 Department of Clinical Sciences and Community Health, University of Milan, 20122 Milan, Italy; 3 Pharmacy Service, Fondazione IRCCS Ca’ Granda Ospedale Maggiore Policlinico (IRCCS), 20122 Milan, Italy; mariagrazia.corti@policlinico.mi.it

**Keywords:** parenteral nutrition weaning, very low birth weight, preterm infants, standardized parenteral nutrition bag, growth velocity, body composition

## Abstract

Weaning from parenteral to enteral nutrition is a critical period to maintain an adequate growth in very low birth weight preterm infants (VLBWI). We evaluated the actual daily nutritional intakes during the transition phase (TP) in VLBWI with adequate and inadequate weight growth velocity (GV ≥ 15 vs. GV < 15 g/kg/day). Fat-free mass (FFM) at term-corrected age (TCA) was compared between groups. Based on actual nutritional intakes of infants with adequate growth, we defined a standardized parenteral nutrition bag (SPB) for the TP. One hundred and six VLBWI were categorized as group 1 (G1): [GV < 15 (n = 56)] and group 2 (G2): [GV ≥ 15 (n = 50)]. The TP was divided into two periods: main parenteral nutritional intakes period (parenteral nutritional intakes >50%) (M-PNI) and main enteral nutritional intakes period (enteral nutritional intakes >50%) (M-ENI). Anthropometric measurements were assessed at discharge and TCA, FFM deposition at TCA. During M-PNI, G2 showed higher enteral protein intake compared to G1 (*p* = 0.05). During M-ENI, G2 showed higher parenteral protein (*p* = 0.01) and energy intakes (*p* < 0.001). A gradual reduction in SPB volume, together with progressive increase in enteral volume, allowed nutritional intakes similar to those of G2. At TCA, G2 had higher FFM compared to G1 (*p* = 0.04). The reasoned use of SPB could guarantee an adequate protein administration, allowing an adequate growth and higher FFM deposition.

## 1. Introduction

The nutritional care of preterm neonates remains a challenge in clinical practice [1,2,3]. Progress has been made to improve the nutrition of very low birth weight preterm infants (VLBWI); however, the transition phase (TP) from parenteral to enteral nutrition still remains a critical period for the achievement of adequate growth [4]. It has been demonstrated that the optimization of nutrition during the TP, as well as maintaining appropriate nutrient intakes, improves growth rates in preterm infants [5,6,7].

Brennan AM et al. derived a target amino-acid intake of 3.5 g/100 mL during TP, using a database of daily nutrient intakes among 59 very low preterm infants [8].

However, the lack of nutritional recommendations during the TP complicates the nutritional management of VLBWI and may contribute to the accretion of nutrients deficit [5]. Consequently, the nutrients deficit may determine poor growth rate and may result in an altered body composition, characterized by a deficit of fat-free mass at term-corrected age (TCA) [9,10]. 

According to these data, the primary aim of this study was to evaluate if an adequate weight growth velocity during the TP is related to an improvement in fat-free mass deposition of VLBWI at TCA.

In addition, the secondary aim was to define an easy-to-use, standardized parenteral nutrition bag (SPB), based on the nutritional intakes of infants with an adequate weight growth velocity during the TP.

## 2. Methods

### 2.1. Study Population

After institutional review board approval (approval code 506_2015), a review of the medical records was conducted on very low birth weight infants (VLBW: birth weight <1500 g) born at the authors’ institution and admitted to the Neonatal Intensive Care Unit from 2015 to 2017. The parents of all the infants included in the study were contacted to obtain their written consent.

The exclusion criteria for all infants screened included congenital diseases, chromosomal abnormalities, cardiac, brain, renal, endocrine or surgical diseases that can interfere with growth and clinical instability at TCA. Specifically, we excluded all participants with ultrasound prenatal or postnatal diagnosis of congenital diseases such as cardiac, brain or renal diseases. Genetic tests were performed when chromosomal abnormalities were suspected. In addition, subjects undergoing major abdominal surgery were excluded, as well as infants with endocrinological diseases confirmed by blood exams.

Gestational age (GA) was calculated based on the last menstrual period and the first trimester ultrasonogram. The corrected age, which is the number of additional weeks from term (40 weeks), was calculated using the chronologic age adjusted for GA [11].

### 2.2. Data Collection and Nutritional Practices

Clinical data, anthropometric measurements and actual daily nutritional intakes in terms of energy [(E): kcal/kg/day] and protein [(P): g/kg/day] were recorded from our nutritional database (Neocare^®^). 

According to our internal procedure, parenteral nutrition (PN) was started on the first day of life. The volume provided was increased from 80–90 mL/kg on the first day up to 150–180 mL/kg on the 7th day of life, with a non-protein energy/protein ratio from 20.8–24 kcal/g on the first day up to 23.1–27.7 kcal/g on the 7th day of life. 

Enteral feeding was started within 24 h of postnatal life using fresh mother’s milk or donor human milk.

When the infants tolerated an enteral intake ≥80 mL/kg, analysis was carried out on the macronutrient content of human milk using a mid-infrared human milk analyzer (MIRIS, Uppsala, Sweden). Then, a target human milk fortification was started using a mono- or polymeric product derived from bovine milk in order to achieve the nutrient intakes recommended by the European Society for Paediatric Gastroenterology Hepatology and Nutrition (ESPGHAN) guidelines [12,13,14]. 

The PN infusion was decreased only in terms of volume, until infants achieved an amount of enteral volume (EV) of at least 50 mL/kg/day. When the enteral feed exceeded 50 mL/kg/day, the PN infusion was progressively decreased.

The TP from parenteral to enteral nutrition was defined as the period between the first day in which the parenteral intakes started to decrease, and the day in which the PN was completely discontinued.

The TP was divided into two main periods: in the first period, the parental nutritional intakes constituted more than 50% of the total (main parenteral nutrition intakes, M-PNI). During the second period, the enteral nutritional intakes constituted more than 50% of the total (main enteral nutrition intakes, M-ENI).

### 2.3. Anthropometric Measurements

Body weight was assessed daily from birth to discharge and at TCA. The body length and head circumference of all infants included in the study were assessed at birth, at discharge and at TCA. All measurements were performed according to standard procedures [15]. In detail, body weight was measured using an electronic scale accurate to the nearest 0.1 g. Body length was measured to the nearest mm using a recumbent infant length board, and head circumference was measured to the nearest 1 mm using a non-stretch measuring tape. The z-scores were computed by using the INTERGROWTH-21st tools [16,17,18].

The weight growth velocity (GV) (g/kg/day) was calculated using an exponential model validated for VLBW infants [19]. The infants included in the study were categorized according to GV during TP in G1: GV < 15 g/kg/day and G2: GV ≥ 15 g/kg/day. A GV ≥ 15 g/kg/day was considered adequate as clinicians commonly use it as a target for weight growth velocity [20].

### 2.4. Body Composition Assessment

Body composition was assessed at TCA for all infants included in the study using an air-displacement plethysmography system (PEA POD Infant Body Composition System; COSMED, Italy) [21,22]. The PEA POD measures both body mass and volume, as well as extrapolating the fat-free mass (FFM) and fat mass (FM) through the application of whole-body densitometric principles. The total absolute (g) and percentage (%) of FFM and FM were calculated by converting body density (derived from the measured mass and volume of the subject) by means of sex-specific equations developed by Fomon et al. in 1982 [23]. The anthropometric and body composition measurements were performed by qualified health specialists, according to standard procedures. The inter-observer coefficient of variation for the FM percentage estimates was 0.3% [24].

### 2.5. Statistical Analysis

Continuous variables are expressed as mean and standard deviation (SD). Categorical variables are described as numbers or percentages. Comparisons between infants that showed GV < 15 g/kg/day during the TP (G1) and infants with GV ≥ 15 g/kg/day (G2) were performed using the X2 test for discrete variables and analysis of variance for continuous variables.

A *p* < 0.05 was considered statistically significant. All statistical analyses were performed by means of SPSS software (SPSS, version 20; SPSS, Chicago, IL, USA).

## 3. Results

During the study period, 1938 infants were admitted to the authors’ institution. Among them, 335 were VLBWI, 42 of whom died during hospitalization while 117 were excluded according to the exclusion criteria. One hundred and seventy-six were eligible infants; among these infants 70 could not be assessed for body composition at term-corrected age. Therefore, 106 infants were included (50% were males and 49% were twins). G1 included 56 infants, whereas 50 infants were categorized in G2. 

The mean birth weight and gestational age were, respectively, 1247 ± 206 g and 30.2 ± 1.9 weeks. No differences in basal characteristics and occurrence of comorbidities were found among the two groups (Table 1 and Table 2). 

The weaning from parenteral nutrition started similarly between groups (16.3 ± 8.3 vs. 14.1 ± 8.2 days of life, respectively, for G1 and G2). The duration of parenteral nutrition (20.9 ± 7.2 and 21.3 ± 8.4 days, respectively, for G1 and G2) and the length of stay (53.2 ± 19.9 and 50.8 ± 18.1 days, respectively, for G1 and G2) were similar among groups.

The total protein and energy intakes during TP were higher in G2 compared to G1 [E: 103.6 ± 16.1 vs. 110.6 ± 15.0 kcal/kg/day (*p* = 0.024) and P: 3.28 ± 0.7 vs. 3.63 ± 0.6 g/kg/day (*p* = 0.006), respectively, for G1 and G2].

During M-PNI, G2 showed a higher enteral protein intake compared to G1.

During M-ENI, G2 showed higher parenteral P and E intakes compared to G1 (Table 3). Specifically during this period, both the parenteral carbohydrate and fat intakes were higher in G2 compared to G1 (carbohydrates: 3.7 ± 2.16 vs. 5.49 ± 2.86 g/kg/day for G1 and G2, respectively; lipids: 1.10 ± 0.64 vs. 1.62 ± 0.84 g/kg/day for G1 and G2, respectively; *p* < 0.001), whereas we did not find any difference during the other period.

No differences in the anthropometric measurements both at discharge and at TCA were detected among the two groups (Table 4).

**Figure 1:**
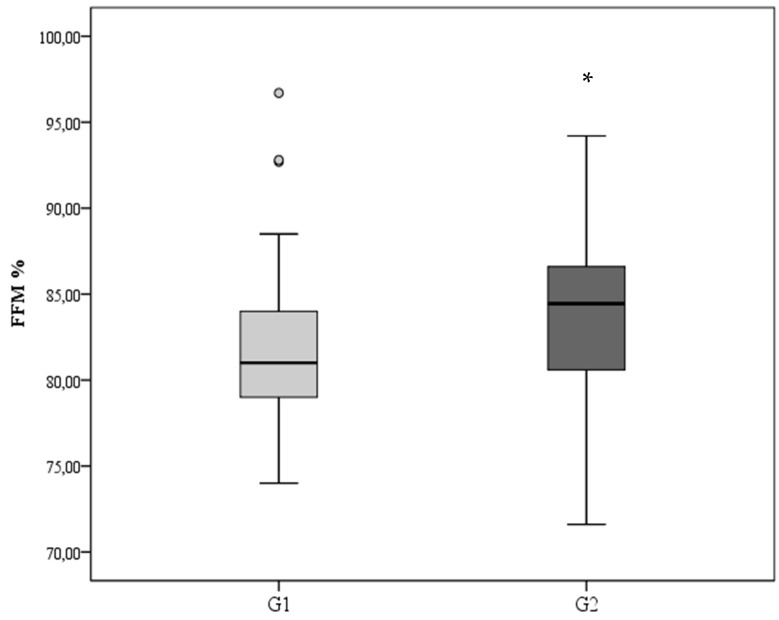
Body composition at TCA in term of Fat Free Mass (FFM) %. * G2 vs. G1; *p* = 0.04.

Despite the similar weight at term-corrected age, at the assessment of the body composition, infants categorized as G2 had higher Fat Free Mass (FFM) compared to G1 (*p* = 0.04, Figure 1).

Considering the actual daily parenteral and enteral intakes of G2, we defined a central line standardized parenteral nutrition bag designed to meet the nutritional needs for preterm infants specifically during the TP. The quality control of the bag was performed by the in-hospital pharmacy service. Table 5 shows the composition of SPB.

**Figure 2:**
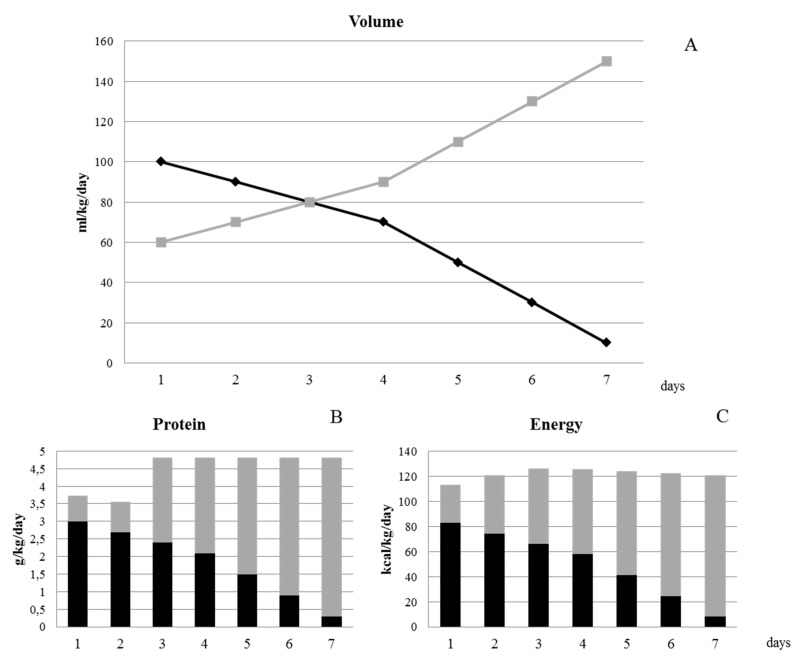
Protocol of weaning from parenteral nutrition. (**A**) The figure shows the progression of the decrease of parenteral nutrition (PN) (black line) and increase of enteral nutrition (light grey line) in terms of volume (mL/kg/day). Figure (**B**) and figure (**C**) show, respectively, the daily protein intakes (g/kg/day) and daily energy intakes (kcal/kg/day) administered by the standard PN bag (black bar) and by enteral nutrition (light grey bar).

Figure 2 illustrates the protocol of weaning from PN using the standard PN bag. The length of weaning is 7 days. Starting from 100 mL/kg/day of PN emulsion and 60 mL/kg/day of enteral nutrition, we suggest an increase of enteral volume intake of 10 mL/kg/day for the first 4 days of TP and of 20 mL/kg/day for the last 3 days, with an equal reduction of the volume of PN. The enteral protein intakes are achieved through target fortification of human milk from the third day of the transition phase (starting from an enteral volume of 80 mL/kg/day).

## 4. Discussion

This study demonstrated that an adequate growth (GV ≥ 15 g/kg/day) during the transition phase is sustained by higher parenteral intakes during the main enteral nutrition phase and is associated to a higher fat-free mass deposition at term-corrected age. Specifically, in the present study we found that during the main PN phase, infants with adequate growth velocity had higher enteral protein intake compared to infants with poor growth velocity, and during the main enteral nutrition phase, they showed higher parenteral protein and energy intakes. The differences in parenteral protein intake during the main enteral nutrition phase were of around 0.5 g/kg/day and the differences in parenteral energy intake were around 10 kcal/kg/day. These differences were comparable to those observed by Miller et al. [7]. 

It has been demonstrated that the transition phase is a critical period to maintain an adequate growth. Indeed, Miller at al. showed that infants with poor growth during the transition phase had a higher risk of having postnatal growth failure at discharge [4]. They supposed that this poor growth could be attributed to the nutrition inadequacy that infants experienced during the TP.

In addition, in a further study, they demonstrated that implementing a nutrition protocol of weaning from parenteral nutrition improved growth in preterm infants [7]. Similar results were obtained in a previous study, performed in our institution, whose aim was to improve the global nutritional approach during the hospital stay [25]. Brennan et al. and Falciglia et al. demonstrated that the evaluation of the actual intakes of nutrients can help to identify the nutritional deficit during the transition phase [5,6].

In a further study, Brennan et al. defined the optimal protein intake during the TP both in the parenteral-dominant period and in the enteral-dominant one [8].

In our study, we also developed an SPB, ready to use, based on the nutritional intakes of those VLBWI that showed an adequate growth rate during the transition phase. The use of this all-in-one SPB provides an appropriate nutritional mixture, and reduces workload and system costs.

The SPB allows us to guarantee the administration of a range of amino acids between 2.5 and 3 g/kg/day during the main parenteral nutrition phase and of 1 to 2 g/kg/day during the main enteral nutrition phase. Actually, in order to reach the amino acid requirements recommended by ESPHGAN during the enteral nutrition phase, the fortification of human milk starting from 80 mL/kg/day is required [6,8,14]. The target fortification that we regularly perform in our institution is a valid instrument to administer the necessary amino acid requirements. 

In the present study, fat-free mass at 40 weeks corrected age was higher in preterm infants with adequate growth velocity during the transition phase than that of preterm infants with lower GV. The FFM values observed in our infants, with adequate growth during the transition phase, were at the 25th percentile of the charts published by Norris et al. [26] and more than the 50th percentile of the charts published by Hamatschek et al. [27].

Concerning body composition, literature data suggest that FFM is possibly related to long-term clinical outcomes. Ramel et al. [28] demonstrated that FFM gain is associated with better neurological and motor outcomes at one and two years corrected age in VLBWI, and Paviotti et al. [29] demonstrated that both fat mass and FFM are related to cerebellar volume at term. Additionally, body composition in early life has been correlated with metabolic disease in adulthood [30]. These data indicate that body composition measurements could be a useful tool to enhance early-life nutritional management growth and long-term outcomes of preterm infants. 

One of the strengths of this study was the large sample size; indeed, we analyzed the actual daily nutrient intakes during the transition period from parenteral to enteral nutrition, recorded in our neonatal nutrition database of 106 preterm infants. Another strength was represented by the fact that we used a target fortification of human milk, which is proved to be a useful tool in order to reach the macronutrient intakes recommended by the ESPGHAN Society. In addition, the body composition measurements, accepted as a useful method to help clinicians optimize the nutritional management and predict long-term outcomes, increased the value of our results.

A limitation of this study was that even where we had defined the prescription of the SPB specific for the transition phase, we did not administer it to our preterm infants.

## 5. Conclusions

The transition phase is a critical period for the growth of preterm infants. The reasoned use of an optimized SPB for the transition phase, associated with target fortified human milk, can be useful for the clinicians in reducing the risk of developing growth retardation in preterm infants.

## Figures and Tables

**Table 1:** Basal characteristics at birth according to weight growth velocity (GV) categorization.

	G1 (GV < 15 g/kg/day)	G2 (GV ≥ 15 g/kg/day)
Gestational age (weeks)	30.1 ± 1.8	30.4 ± 1.9
Weight (g)	1222 ± 199	1274 ± 213
Length (cm)	37.7 ± 2.9	37.8 ± 2.1
Head circumference (cm)	26.5 ± 2.2	26.9 ± 2.0
Weight Z-score	−0.59 ± 1.1	−0.76 ± 0.9
Length Z-score	−0.86 ± 1.1	−1.07 ± 1.3
Head circumference Z-score	−0.55 ± 1.2	−0.51 ± 0.9

All data are expressed as mean ± SD.

**Table 2:** Occurrence of comorbidities.

	G1 (GV < 15 g/kg/day)	G2 (GV ≥ 15 g/kg/day)
Necrotizing enterocolitis	2 (3.6)	1 (2)
Cholestasis	6 (10.7)	3 (6)
Bronchopulmonary dysplasia	1 (1.8)	2 (4)
Abdominal surgery	3 (5.3)	1 (2)
Patent ductus arteriosus	2 (3.6)	3 (6)
Retinopathy of prematurity	3 (5.3)	0 (0)
Osteopenia	4 (7.1)	5 (10)
Intraventricular hemorrhage	2 (3.6)	1 (2)
Sepsis	7 (12.5)	8 (16)

All data are expressed as absolute numbers and percentages (%).

**Table 3:** Nutritional intakes during the transition phase (TP) from parenteral to enteral nutrition during the mean parenteral nutrition intake period (parenteral nutrition intake >50%; M-PNI) and during the mean enteral nutrition intake period (enteral nutrition intake >50%; M-ENI).

		G1 (GV < 15 g/kg/day)	G2 (GV ≥ 15 g/kg/day)	*p*
M-PNI	Parenteral protein intake (g/kg/day)	2.36 ± 0.7	2.45 ± 0.8	0.53
	Parenteral energy intake (Kcal/kg/day)	59.4 ± 18.6	61.9 ± 24.1	0.54
	Enteral protein intake (g/kg/day)	1.09 ± 0.5	1.33 ± 0.7	0.05
	Enteral energy intake (Kcal/kg/day)	45.5 ± 16.4	51.5 ± 21.3	0.10
M-ENI	Parenteral protein intake (g/kg/day)	1.28 ± 0.7	1.74 ± 0.9	0.01
	Parenteral energy intake (Kcal/kg/day)	30.0 ± 17.0	43.6 ± 22.6	<0.001
	Enteral protein intake (g/kg/day)	1.82 ± 0.8	1.73 ± 0.8	0.58
	Enteral energy intake (Kcal/kg/day)	72.4 ± 20.0	64.2 ± 26.5	0.07

All data are expressed as mean ± SD.

**Table 4:** Anthropometric measurements at discharge and at term corrected age (TCA).

		G1 (GV < 15 g/kg/day)	G2 (GV ≥ 15 g/kg/day)
Discharge	Weight (g)	2438 ± 441	2505 ± 453
	Length (cm)	45.2 ± 2.5	45.5 ± 2.5
	Head circumference (cm)	32.3 ± 1.5	32.3 ± 1.4
	Weight Z-score	−1.02 ± 1.1	−0.82 ± 1.1
	Length Z-score	−1.72 ± 1.6	−1.26 ± 1.2
	Head circumference Z-score	−1.45 ± 1.3	−0.95 ± 1.3
TCA	Weight (g)	3207 ± 464	3220 ± 533
	Length (cm)	48.5 ± 2.0	48.7 ± 2.5
	Head circumference (cm)	34.9 ± 1.3	34.9 ± 1.4

All data are expressed as mean ± standard deviation.

**Table 5:** Standard parenteral nutrition bag.

Parenteral Nutrition Mixtures Bag “All-in-One” Quantity /100 mL	
Amino-acids 10% (g)	3
Glucose (g)	14
Lipids (20%) (g)	3
Sodium (mmol)	3
Potassium (mmol)	3
Calcium (mmol)	1.6
Phosphate (mmol)	1.6
Oligoelements (mL)	1
Hydrosoluble vitamins (mL)	1
Liposoluble vitamins (mL)	4
Osmolarity (mOsm/L)	1141
